# Facial cues of dominance modulate the short-term gaze-cuing effect in human observers

**DOI:** 10.1098/rspb.2009.1575

**Published:** 2009-10-28

**Authors:** Benedict C. Jones, Lisa M. DeBruine, Julie C. Main, Anthony C. Little, Lisa L. M. Welling, David R. Feinberg, Bernard P. Tiddeman

**Affiliations:** 1 School of Psychology, University of Aberdeen, Scotland, UK; 2 School of Psychology, University of Stirling, Scotland, UK; 3 Department of Psychology, Neuroscience and Behaviour, McMaster University, Canada; 4 School of Computer Science, University of St Andrews, Scotland, UK

**Keywords:** gaze, dominance, gaze-following, joint attention

## Abstract

Responding appropriately to gaze cues is essential for fluent social interaction, playing a crucial role in social learning, collaboration, threat assessment and understanding others’ intentions. Previous research has shown that responses to gaze cues can be studied by investigating the gaze-cuing effect (i.e. the tendency for observers to respond more quickly to targets in locations that were cued by others’ gaze than to uncued targets). A recent study demonstrating that macaques demonstrate larger gaze-cuing effects when viewing dominant conspecifics than when viewing subordinate conspecifics suggests that cues of dominance modulate the gaze-cuing effect in at least one primate species. Here, we show a similar effect of facial cues associated with dominance on gaze cuing in human observers: at short viewing times, observers demonstrated a greater cuing effect for gaze cues from masculinized (i.e. dominant) faces than from feminized (i.e. subordinate) faces. Moreover, this effect of facial masculinity on gaze cuing decreased as viewing time was increased, suggesting that the effect is driven by involuntary responses. Our findings suggest that the mechanisms that underpin reflexive gaze cuing evolved to be sensitive to facial cues of others’ dominance, potentially because such differential gaze cuing promoted desirable outcomes from encounters with dominant individuals.

## 1. Introduction

The ability to follow others’ gaze is important for social interaction in many species, playing a critical role in collaboration, social learning, threat assessments and understanding others’ intentions and attitudes (Baron-Cohen 1995; Emery 2000; Tomasello *et al*. 2005; Zuberbuhler & Byrne 2006; Frischen *et al*. 2007; Frith & Frith 2007; Zuberbuhler 2008). Indeed, gaze-following is thought to occur in most primate species, from prosimians to humans (Zuberbuhler 2008). Responses to gaze cues have been most extensively researched in humans (see Frischen *et al*. 2007 for a review), other great apes (e.g. Tomasello *et al*. 1999; Brauer *et al*. 2005), and macaques (*Macaca mulatta*, e.g. Emery *et al*. 1997; Ferrari *et al*. 2000; Deaner & Platt 2003; Shepherd *et al*. 2006). However, the capacity for gaze-following is by no means limited to primates. For example, gaze-following has also been reported in dogs (*Canis familiaris*), goats (*Capra hircus*), ravens (*Corvus corax*), bottlenose dolphins (*Tursiops truncates*) and fur seals (*Arctocephalus pusillus*, Tschudin *et al*. 2001; Hare *et al*. 2002; Bugnyar *et al*. 2004; Scheumann & Call 2004; Kaminski *et al*. 2005; Schloegl *et al*. 2007).

Responses to gaze cues in humans are most commonly studied using variations of Posner's spatial cuing paradigm (Posner 1980; Posner & Cohen 1984). In this paradigm, the gaze direction of a centrally presented face image can be either congruent or incongruent with the location of a subsequently presented target. Studies using this paradigm have shown that human observers tend to be faster to respond to targets presented in gaze-congruent locations than to targets presented in gaze-incongruent locations, a phenomenon that is often referred to as the *gaze-cuing effect* (e.g. Driver *et al*. 1999; Langton & Bruce 1999; Deaner & Platt 2003; Hietanen & Leppanen 2003; Shepherd *et al*. 2006; Deaner *et al*. 2007). Importantly, this gaze-cuing effect in human observers occurs at short *viewing times* (e.g. 300 ms) even when gaze cues are counterpredictive (Driver *et al*. 1999). By contrast, no such gaze-cuing effect appears to occur at long viewing times (e.g. 800 ms) under these circumstances (Driver *et al*. 1999). Collectively, these findings suggest a reflexive (i.e. involuntary) component to the gaze-cuing effect that is most apparent at short viewing times (Driver *et al*. 1999; see also Friesen & Kingstone 1998; Langton & Bruce 1999; Deaner & Platt 2003). The absence of a comparable cuing effect at longer viewing times is thought to reflect the involuntary component of this short-term cuing effect having occurred *before* the target stimulus is presented, coupled with the well-established tendency for observers to demonstrate reduced attention to locations that were recently inspected (Friesen & Kingstone 1998; Driver *et al*. 1999; Langton & Bruce 1999). Related studies of responses to gaze cues in macaques have also implicated an involuntary component that is most apparent at short viewing times (e.g. Deaner & Platt 2003).

Many researchers have emphasized that, in humans at least, the gaze-cuing effect appears to be generally unaffected by *facial* cues other than gaze direction (e.g. Hietanen & Leppanen 2003; Bayliss *et al*. 2007; Frischen *et al*. 2007). Indeed, a recent review of the literature on gaze cuing in humans concluded that ‘changing perceptual or semantic properties of the face stimulus does not appear to affect the short-term gaze-cuing effect in the general population’ (Frischen *et al*. 2007, p. 709). This conclusion was largely based on studies in which the short-term gaze-cuing effect was unaffected by familiarity with the individuals presented (Frischen & Tipper 2004) or their facial expressions (e.g. Hietanen & Leppanen 2003; Bayliss *et al*. 2007). However, and as Frischen *et al*. (2007) acknowledged, some studies of gaze cuing in humans have presented evidence that facial cues other than gaze direction can modulate the short-term gaze-cuing effect under certain conditions. For example, some studies observed a greater gaze-cuing effect when viewing faces with fearful expressions than when viewing faces with other expressions when dynamic changes in gaze direction and facial expression occur simultaneously (Tipples 2006), when positively and negatively valenced targets are used (Pecchinenda *et al*. 2008), or among observers who report high levels of anxiety (Mathews *et al*. 2003; Putman *et al*. 2006; Fox *et al*. 2007, see also Holmes *et al*. (2006) for a similar effect of anxiety for both angry and fearful facial expressions). Although some previous studies have found that facial expressions modulate responses to gaze cues in macaques (Goossens *et al*. 2008), other studies found no effect of facial expressions on gaze-following in macaques (Paukner *et al*. 2007). In humans, greater gaze-cuing effects have also been observed for personally familiar individuals than for unfamiliar individuals, although this effect of familiarity was only evident in female participants (Deaner *et al*. 2007). Collectively, these findings suggest that facial cues other than gaze direction can modulate the short-term gaze cuing effect in human observers under some circumstances.

A recent study by Shepherd *et al*. (2006) found that male macaques demonstrated greater gaze-cuing effects when observing dominant males than when observing subordinate males. Thus, dominance appears to modulate gaze cuing in macaques, potentially reflecting the effects of facial cues associated with dominance (Deaner *et al*. 2007). However, it is not known whether cues of dominance affect gaze cuing in other primate species, including humans. Facial cues associated with dominance might be expected to affect responses to gaze cues in macaque and human observers in similar ways, given that the temporal and spatial dynamics of gaze cuing in humans and macaques are virtually identical (Deaner & Platt 2003) and because the neurobiological bases of gaze cuing in these species are also very similar (Zuberbuhler 2008). Moreover, Oosterhof & Todorov (2008) recently demonstrated that perceived facial dominance is a particularly important trait for sociocognitive processing of faces in humans. Indeed, ratings of facial dominance are positively associated with men's social status (Mueller & Mazur 1996) and upper body strength (Fink *et al*. 2007; see also Sell *et al*. 2009), suggesting that perceptions of facial dominance in humans are somewhat accurate (Mueller & Mazur 1996; Fink *et al*. 2007). Greater gaze cuing for human faces displaying cues associated with high dominance than for faces displaying cues associated with low dominance in human observers would present novel evidence for dominance-contingent gaze cuing in primates and would suggest that the short-term gaze-cuing effect in human observers is sensitive to facial cues other than gaze direction.

Many studies have reported very strong positive relationships between masculine facial features and the perceived dominance of men and women (Perrett *et al*. 1998; DeBruine *et al*. 2006; Boothroyd *et al*. 2007; Fink *et al*. 2007; Conway *et al*. 2009; Main *et al*. 2009). Thus, we compared the gaze-cuing effect when human observers viewed human face images that were either masculinized or feminized using well-established computer graphic methods that have been used to manufacture stimuli in many previous studies of face perception (e.g. Perrett *et al*. 1998; Penton-Voak *et al*. 1999; DeBruine *et al*. 2006). Given Shepherd *et al*.'s (2006) findings for gaze cuing and dominance in macaques, we predicted that the gaze-cuing effect in human observers would be greater on trials where gaze cues were provided by masculine (i.e. dominant) faces than on trials where gaze cues were provided by relatively feminine (i.e. subordinate) faces. We tested for such a masculinity-contingent gaze-cuing effect at three different viewing times (200, 400, 800 ms) in order to investigate whether the predicted effect of masculinity reflects involuntary (i.e. reflexive) responses or voluntary (i.e. deliberate) responses. As previous studies have shown that the short-term reflexive component of gaze cuing is apparent at short viewing times, but not at long viewing times (Driver *et al*. 1999; see also Friesen & Kingstone 1998; Langton & Bruce 1999; Deaner & Platt 2003), an effect of facial masculinity on the gaze-cuing effect at short viewing times, but not long viewing times, would implicate reflexive responses in masculinity-contingent gaze cuing. By contrast, an effect of facial masculinity on gaze cuing at long viewing times, but not short viewing times, would suggest that masculinity-contingent gaze cuing was primarily driven by voluntary responses. As discussed previously, the tendency to demonstrate decreased attention to locations that were recently inspected is thought to be a direct consequence of the reflexive nature of the short-term gaze-cuing effect (Friesen & Kingstone 1998; Driver *et al*. 1999; Langton & Bruce 1999; Deaner & Platt 2003). Thus, if facial masculinity facilitates reflexive responses to gaze cues, increasing viewing time would also be expected to decrease the gaze-cuing effect for masculine faces, but not necessarily for feminine faces. As previous studies found no effects of the sex of face or the sex of participant on the perceived dominance of masculinized versus feminized faces (Perrett *et al*. 1998; Main *et al*. 2009), we anticipated that the predicted masculinity-contingent gaze-cuing effect would not be qualified by the sex of the face presented or the sex of the observer.

## 2. Material and Methods

### (a) Stimuli

**Figure 1.**
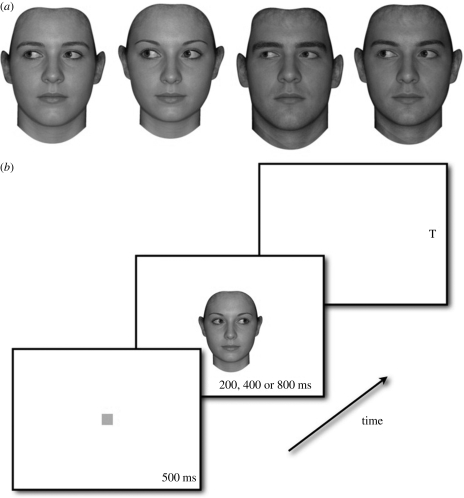
(*a*) Masculinized (leftmost faces in the male and female pairs) and feminized (rightmost faces in the male and female pairs) prototype faces used in our experiment. (*b*) The gaze-cuing task. The figure shows an example of a trial where gaze direction and target location are congruent.

Following many previous studies of the effects of masculinity–femininity on face processing (e.g. Perrett *et al*. 1998; Penton-Voak *et al*. 1999; DeBruine *et al*. 2006; Conway *et al*. 2009; Main *et al*. 2009), we used prototype-based image transformations to objectively and systematically manipulate masculinity–femininity of two-dimensional shape in prototype faces (figure 1*a*). Using prototype faces as stimuli ensures that the masculinized and feminized versions are more masculine and feminine than average and ensures that our face stimuli are highly representative (i.e. prototypic) of the intended categories (Perrett *et al*. 1998; Penton-Voak *et al*. 1999). Only sexually dimorphic shape cues are altered using these methods (Perrett *et al*. 1998; Penton-Voak *et al*. 1999); colour and texture cues are unaltered.

First, we manufactured male and female prototype (i.e. average) faces with averted gaze by averaging the shape, colour and texture information from images of 24 young adult men (to manufacture the male prototype) and 24 young adult women (to manufacture the female prototype) who were photographed with gaze averted to the left. Technical details of the computer graphic methods used to manufacture these prototypes are given in Tiddeman *et al*. (2001).

Next, we manufactured masculinized and feminized versions of the averted gaze composites by applying plus or minus 75 per cent of the vector differences in two-dimensional shape between symmetrized male and female prototypes with direct gaze to the male and female prototypes with averted gaze. We used the difference between male and female prototypes with direct gaze to masculinize and feminize face shape to ensure that masculinizing and feminizing the averted gaze images did not alter gaze direction. Technical details of the computer graphic methods used to transform two-dimensional face shape in this way are given in Tiddeman *et al*. (2001) and Perrett *et al*. (1998).

Finally, each of the masculinized and feminized versions of the prototypes with gaze averted to the left was mirror-reversed around their central vertical axis to create corresponding images in which the gaze was averted to the right. The masculinized and feminized male and female faces were masked so that hairstyle and clothing were not visible. Note that our stimuli were the masculinized and feminized versions of a male prototype face and a female prototype face, and not the masculinized and feminized versions of individuals. Previous research on the gaze-cuing effect (Bayliss *et al*. 2005) and perceptions of masculinized versus feminized faces (Perrett *et al*. 1998) has also used a single image for each face category.

### (b) Manipulation check

Participants (*N* = 60, 26 women, 34 men; all aged between 18 and 38 years) were shown the pairs of faces with averted gaze (each pair consisting of masculinized and feminized versions of the same prototype with gaze averted in the same direction) in a fully randomized order and were asked to indicate which individual was more masculine (20 participants), more dominant (20 participants) or physically stronger (20 participants). The side of the screen on which any particular image was shown was fully randomized. For each combination of judgement type (masculinity, dominance, physical strength) and sex of face (male, female), participants were more likely to choose the masculinized version than the feminized version (binomial tests: all *p* < 0.01, proportion of subjects choosing masculinized faces all greater than 0.85), demonstrating that masculinized prototypes were perceived to be more masculine, dominant and physically stronger than feminized individuals.

### (c) Participants

Ten male and 10 female observers (mean age = 31.15 yr, s.d. = 7.94 yr) participated in the gaze-cuing task. The number of participants in our study is equivalent to or greater than the sample size in many previous studies of gaze cuing (e.g. Driver *et al*. 1999). Hietanen & Leppanen (2003) have previously shown that findings for gaze cuing in samples of this size generalize to much larger samples.

### (d) Procedure

The gaze-cuing task we used (figure 1*b*) is based on those used in Deaner *et al*. (2007) and Driver *et al*. (1999). On each trial, observers initially fixated on an orange square (1.3°, i.e. 1.3 degrees of visual angle) at the centre of the screen for 500 ms. This fixation object was then replaced with a face image (7.6°) with left or right averted gaze. This face image was presented at the centre of the screen and was a masculinized male, feminized male, masculinized female or feminized female prototype. The face image disappeared after 200, 400, or 800 ms viewing time and a peripherally located target (either an uppercase L or uppercase T approx. 1.3° in size) was immediately presented on either the left or the right of the screen. Left and right targets were symmetrically located 12.5° from the centre of the screen and could be either congruent or incongruent with the gaze cue (i.e. could appear on the side of the screen cued by the gaze direction of the preceding image or could appear on the side of the screen that was not cued by the gaze direction of the preceding image).

On each trial, the observer was instructed to indicate as quickly and accurately as possible whether the target was an L or a T. Following Langton & Bruce (1999), participants were told to ignore the face and that gaze cues did not usefully predict the probable location of the target. Responses were made by pressing the 1 or 7 keys on a numberpad with the index finger on the dominant hand. Note that the manual responses, up and down, were dissociated from the possible target locations, left and right (following, e.g. Driver *et al*. 1999; Deaner *et al*. 2007). Half of the participants (five male and five female) used the 1 key to indicate that the target was an L and the 7 key to indicate that the target was a T. The other half of the participants (five male and five female) used the 1 key to indicate that the target was a T and the 7 key to indicate that the target was an L. The target remained onscreen until a response was made or 1200 ms elapsed.

Each observer completed 768 trials, in which face type (masculinized, feminized), face sex (male, female), viewing time (200, 400 and 800 ms), location of target (left, right), congruency of gaze cue (congruent, incongruent) and the type of target (T or L) were fully counterbalanced. Trials were split into eight blocks of 96 trials, each block containing an equal number of each combination of face type, face sex, viewing time, location of target, congruency of gaze cue and the type of target. Trial order was fully randomized in each block.

The eight blocks of experimental trials were preceded by 40 practice trials.

### (e) Initial processing of data

Following Deaner *et al*. (2007), we excluded trials where incorrect responses were given, responses preceded the target presentation, the response time was greater than three standard deviations above or below each observer's overall mean, or no response was made within 1200 ms of the target appearing (see also Driver *et al*. 1999). This process excluded less than 5 per cent of trials in total. The mean response time was 526.92 ms (s.d. = 64.85 ms).

We calculated the mean response time for gaze-congruent and gaze-incongruent trials in each condition for each observer. For each observer, we then calculated the gaze-cuing effect for each condition by subtracting the mean response time for gaze-congruent trials from the mean response time for gaze-incongruent trials (following, e.g. Deaner *et al*. 2007). The mean gaze-cuing effect and SEMs for each condition are given in table 1. Kolmogorov–Smirnov tests showed that all of these scores were normally distributed (all *Z* < 0.82, all *p* > 0.51).

**Table 1.** The mean gaze-cuing effect (ms) in each condition for male and female observers. SEMs are given in parentheses.

viewing time (ms)	observer sex	masculinized male face	feminized male face	masculinized female face	feminized female face
200	male	19.42 (6.24)	12.84 (12.47)	12.38 (5.53)	−6.5 (8.5)
200	female	23.41 (7.61)	8.85 (8.78)	10.64 (6.19)	6.93 (5.33)
400	male	7.17 (5.89)	−5.28 (6.34)	−2.13 (8.34)	13.01 (6.11)
400	female	13.03 (9.17)	3.67 (8.00)	13.73 (6.35)	0.20 (7.00)
800	male	8.65 (6.90)	18.45 (8.09)	3.82 (6.08)	7.65 (6.26)
800	female	0.77 (5.89)	0.65 (7.26)	5.19 (9.22)	16.11 (8.30)

We also calculated the percentage of discrimination errors after excluding trials where responses preceded the target presentation, the response time was greater than three standard deviations above or below each observer's overall mean, or no response was made within 1200 ms of the target appearing. As for the response time data, for each observer, we calculated the gaze-cuing effect on error rates for each condition by subtracting the mean error rate for gaze-congruent trials from the mean error rate for gaze-incongruent trials.

## 3. Results

### (a) Response times

**Figure 2.**
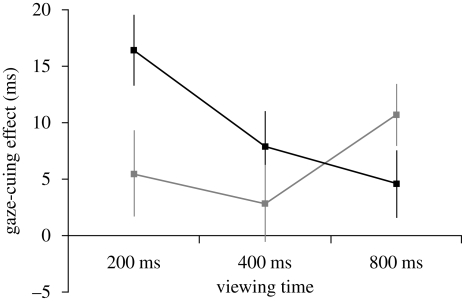
The significant interaction between the effects of face type and viewing time on gaze cuing. Squares show the mean gaze-cuing effect (ms) for each condition and error bars show s.e.m. The gaze-cuing effect for masculinized faces was significantly greater than that for feminized faces at the 200 ms viewing time, but not at the 400 or 800 ms viewing times, suggesting that masculinity influences reflexive short-term cuing. Consistent with this proposal, significant gaze-cuing effects for masculinized faces were observed at the 200 and 400 ms viewing times, but not at the 800 ms viewing time and there was a significant linear effect of viewing time on gaze cuing for masculinized faces. By contrast, there was a significant gaze-cuing effect for feminized faces at the 800 ms viewing time only and the linear effect of viewing time on gaze cuing for feminized faces was not significant. Black squared line, masculinized faces; grey squared line, feminized faces.

Gaze-cuing effects were first analysed using a mixed design ANOVA (within-subjects factors: sex of face (male, female), viewing time (200, 400 and 800 ms), face type (masculinized, feminized); between subjects factor: sex of observer (male, female)). Tests for within-subjects and between-subjects effects revealed the predicted interaction between face type and viewing time (*F*(2,36) = 4.74, *p* = 0.015, partial η^2^ = 0.21, see figure 2) and no other significant effects (all *F* < 1.2, all *p* > 0.290, all partial η^2^ < 0.07). Tests for within-subjects polynomial contrasts revealed the predicted linear interaction between face type and viewing time (*F*(1,18) = 8.47, *p* = 0.009, partial η^2^ = 0.32, see figure 2) and no other linear effects (all *F* < 1.2, all *p* > 0.290, all partial η^2^ < 0.07).

To interpret the interaction between face type and viewing time that was revealed by the tests for within-subjects effects, we conducted planned comparisons using paired-samples *t*-tests. These planned comparisons showed that the gaze-cuing effect was significantly greater for masculinized faces than for feminized faces at the 200 ms viewing time (*t*(19) = 2.18, *p* = 0.042, *d* = 0.49), but not at the 400 ms (*t*(19) = 1.08, *p* = 0.292, *d* = 0.24) or 800 ms (*t*(19) = −1.72, *p* = 0.102, *d* = 0.39) viewing times.

To interpret the linear interaction between face type and viewing time that was revealed by the tests for within-subjects polynomial contrasts, we repeated the initial ANOVA for masculinized and feminized faces separately. The within-subjects contrasts showed a significant linear effect of viewing time for masculinized faces (*F*(1,19) = 5.46, *p* = 0.031, partial η^2^ = 0.22), but not for feminized faces (*F*(1,19) = 1.18, *p* = 0.291, partial η^2^ = 0.06), as we had predicted.

Finally, we used one-sample *t*-tests to compare the gaze-cuing effect in each condition with what would be expected if there were no gaze-cuing effect (i.e. the chance value of 0 ms). Data were collapsed across the factor sex of face because the previous ANOVA did not reveal any effects of sex of face. The one-sample *t*-tests showed that participants were faster to respond to gaze-congruent targets than to gaze-incongruent targets for trials on which masculinized faces were shown for 200 ms (*t*(19) = 5.36, *p* < 0.001, *d* = 1.20) and 400 ms (*t*(19) = 2.56, *p* = 0.019, *d* = 0.57), but not for trials on which masculinized faces were shown for 800 ms (*t*(19) = 1.57, *p* = 0.133, *d* = 0.35). These analyses also showed that participants were faster to respond to gaze-congruent targets than to gaze-incongruent targets for trials on which feminized faces were shown for 800 ms (*t*(19) = 3.89, *p* < 0.001, *d* = 0.87), but not for trials on which feminized faces were shown for 200 ms (*t*(19) = 1.47, *p* = 0.157, *d* = 0.32) or 400 ms (*t*(19) = 0.87, *p* = 0.397, *d* = 0.19).

### (b) Error rates

We used a mixed design ANOVA to compare the effects of gaze cuing on error rates in each condition (within-subjects factors: sex of face (male, female), viewing time (200, 400 and 800 ms), face type (masculinized, feminized); between subjects factor: sex of observer (male, female)). The dependent variable was calculated by subtracting the error rate for gaze-congruent trials from the error rate for gaze-incongruent trials separately for each participant and each condition. This analysis revealed a significant main effect of sex of observer (*F*(1, 18) = 4.96, *p* = 0.039, partial η^2^ = 0.22), whereby women were less probable to make errors when discriminating between targets in gaze-congruent locations than when discriminating between targets in gaze-incongruent locations (*t*(9) = −3.91, *p* = 0.004, *d* = 1.24), but men were not (*t*(9) = −0.09, *p* = 0.99, *d* = 0.03). There were no other effects (all *F* < 1.80, all *p* > 0.18, all partial η^2^ < 0.09). That female observers demonstrated a greater gaze-cuing effect on error rates than male observers did is consistent with previous research in which gaze-cuing effects on response times were greater for human female observers (Bayliss *et al*. 2005) and in which female pigtailed macaques were more likely to follow cues to the direction of others’ attention than male macaques were (Paukner *et al*. 2007). Although Khurana *et al*. (2009) has recently reported that human observers showed greater gaze-cuing effects for opposite-sex faces than for own-sex faces, no such opposite-sex bias was evident in our study.

## 4. Discussion

Analyses showed that the gaze-cuing effect was significantly greater for masculinized faces than for feminized faces at the shortest viewing time (i.e. 200 ms), but not at longer viewing times. Moreover, significant gaze-cuing effects were observed at short viewing times (i.e. 200 and 400 ms) for masculinized faces, but not for feminized faces. As previous studies have demonstrated that the reflexive component of the short-term gaze-cuing effect is most apparent at short viewing times (e.g. Driver *et al*. 1999; see also Friesen & Kingstone 1998; Langton & Bruce 1999; Deaner & Platt 2003), our findings suggest that facial masculinity modulates reflexive gaze cuing. Additionally, as decreased gaze cuing at longer viewing times is thought to be a direct consequence of the reflexive component of the short-term gaze-cuing effect (Friesen & Kingstone 1998; Driver *et al*. 1999; Langton & Bruce 1999; Deaner & Platt 2003), the linear effect of viewing time that we observed for masculinized faces, but not for feminized faces, also suggests that facial masculinity modulates the reflexive component of the short-term gaze-cuing effect. It is well established that masculinized faces are perceived to be more dominant than feminized faces (Perrett *et al*. 1998; DeBruine *et al*. 2006; Boothroyd *et al*. 2007; Main *et al*. 2009). Consistent with these findings, the manipulation check that we conducted in the current study demonstrated that our masculinized faces were perceived both as more dominant and as physically stronger than our femininized faces. Modulation of the short-term gaze-cuing effect by facial masculinity among human observers then complements dominance-contingent gaze cuing previously reported in macaques (Shepherd *et al*. 2006). That the effect of masculinity on gaze cuing in our study was not qualified by interactions with either the sex of observer or the sex of face presented is consistent with previous studies that found no effects of sex of face or sex of participant on the perceived dominance of masculinized versus feminized faces (Perrett *et al*. 1998; Main *et al*. 2009).

Previous findings for differential gaze cuing according to facial cues other than gaze direction in human observers have been somewhat mixed. For example, while some studies have observed effects of facial expression under certain circumstances (e.g. among anxious individuals, Mathews *et al*. 2003), others have observed no effect of facial expression on gaze cuing in the general population (e.g. Hietanen & Leppanen 2003; Bayliss *et al*. 2007). The latter findings have led many researchers to conclude that, for human observers, the short-term gaze-cuing effect in the general population is relatively unaffected by facial cues other than gaze direction (see Frischen *et al*. 2007 for a review). By contrast with this conclusion, however, our findings suggest that facial cues associated with dominance can modulate the short-term gaze-cuing effect in human observers, suggesting that studies of gaze cuing in humans can profit by considering findings for gaze cuing in other primate species. Additionally, both our findings for masculinity and gaze cuing in human observers and Shepherd *et al*.'s (2006) findings for dominance and gaze cuing in macaques suggest that further research exploring dominance-contingent gaze cuing in other social species may prove fruitful.

That previous studies have found no significant difference between the short-term gaze-cuing effects for angry and fearful facial expressions in human observers (e.g. Mathews *et al*. 2003)^1^ helps clarify the aspect of dominance that may be important for greater gaze cuing at short viewing times for masculinized faces than for feminized faces. Angry faces are typically rated as more dominant than fearful faces (Hess *et al*. 2000). That we observed greater gaze cuing for masculinized (i.e. dominant) faces than for feminized (i.e. subordinate) faces therefore raises the question of why greater gaze cuing does not appear to occur for angry than for fearful faces. Angry and fearful facial expressions reflect rapid, relatively brief, dynamic changes in facial appearance that are typically produced in response to external stimuli. As such, in isolation, they provide little information about an individual's position in dominance hierarchies (i.e. their rank). By contrast, masculine characteristics are a relatively invariant physical aspect of facial appearance that are far more stable over time and different social settings and that are known to be associated both with social status in modern human societies (Mueller & Mazur 1996) and with traits that would have presumably been important factors for social rank in ancestral times and in groups of non-human primates (e.g. physical strength, Fink *et al*. 2007; Sell *et al*. 2009). Thus, we suggest that the effect of facial masculinity on gaze cuing observed in our experiment is more likely to reflect the association between the physical aspects of facial appearance and probable dominance rank than an association between perceived dominance and transient (i.e. dynamic) aspects of facial appearance, such as has been observed for angry facial expressions. This interpretation is consistent with Deaner *et al*.'s (2007) suggestion that facial dominance mediates the effect of actual social status on gaze cuing in macaques. Indeed, while Fox *et al*. (2007) found that angry facial expressions did not have a significant effect on gaze cuing in human observers, angry faces with direct gaze captured observers’ attention more than faces showing other emotional expressions. Thus, Fox *et al*.'s (2007) results are consistent with our suggestion that, while angry facial expressions signal information that is clearly important for perceptual and behavioural responses, the information that is signalled by angry faces appears to be qualitatively different to that which modulates gaze cuing.

Intriguingly, while cues associated with dominance modulated the reflexive component of the short-term gaze-cuing effect in the current study, the dominance of the faces presented appears to modulate a later (i.e. volitional) component of gaze cuing in macaques (Shepherd *et al*. 2006). Identifying whether this difference in the time course and nature of dominance-contingent gaze cuing in humans and macaques reflects subtle differences in the qualities signalled by facial cues of dominance in each species, differences in the methodologies employed in these studies (e.g. the use of familiar versus unfamiliar individuals as stimuli or the use of actual versus perceptual measures of dominance), or a combination of such factors, may provide further insight into the differences and similarities in responses to social gaze across species.

We created masculinized and feminized faces for our experiment by varying face shape along a dimension that was defined by the linear differences between symmetric male and female prototypes (see also Perrett *et al*. 1998; Penton-Voak *et al*. 1999). This means that our feminized face stimuli have slightly larger eyes than our masculinized stimuli because female faces have larger eyes than male faces do (e.g. Penton-Voak *et al*. 2001 for facial-metric evidence). Consequently, we suggest that the observed effect of masculinized versus feminized face shape on gaze cuing is unlikely to reflect the effects of simple physical (i.e. low-level) differences between the eye regions of masculinized and feminized faces that are known to affect gaze processing (e.g. differences in contrast and luminance distribution, Sinha 2000; Ricciardelli *et al*. 2000; Ando 2004). To elaborate, the larger area of visible sclera in feminized faces should facilitate, rather than impede, gaze processing. This suggests that the facilitating effect of masculine configural shape cues on the gaze-cuing effect is greater than the potentially impeding effect owing to the differences in low-level features in the eye region. While our findings suggest that masculinity-contingent gaze cuing in human observers is unlikely to simply be a consequence of inflexible responses to low-level properties of the eye region and implicates a role for high-level facial properties in differential gaze cuing, we acknowledge that more direct tests of this proposal are needed to clarify this issue. One possible direction for future research on this topic would be to investigate whether perceptions of others’ dominance directly mediate masculinity-contingent gaze cuing. Similarly, one could also test whether indices of actual physical dominance, such as measures of physical strength (Fink *et al*. 2007; Sell *et al*. 2009), mediate the differential response to social gaze that was observed in the current experiment. We suggest that these issues are important topics for future research. We also suggest that identifying whether the effect of facial cues of dominance on gaze cuing in human observers occurs only for covert shifts in visual attention or also extends to overt eye movements is an interesting topic for future research.

In summary, we show that facial cues associated with dominance (masculinity of face shape) modulate the short-term gaze-cuing effect in human observers. Observers demonstrated greater gaze cuing for masculinized faces than for feminized faces at short viewing times, but not at long viewing times, implicating reflexive responses in masculinity-contingent gaze cuing. These findings complement a previously reported effect of dominance on gaze cuing in a non-human primate species (macaque monkeys, Shepherd *et al*. 2006). A shared mechanism could suggest that dominance-contingent gaze cuing arose early in the primate lineage and may have been present in a common ancestor of the two species prior to their divergence. Collectively, these findings for dominance and gaze cuing in humans and macaques suggest that the mechanisms and processes that underpin responses to social gaze evolved to be sensitive to cues of dominance, potentially because such sensitivity would promote fluent social interactions with dominant individuals and, ultimately, desirable social outcomes.

## Endnote

1 Some studies have observed greater gaze cuing for fearful faces than angry faces, however, though this effect only occurred among highly anxious observers (Fox *et al*. 2007).
